# Normative productivity of the global vegetation

**DOI:** 10.1186/1750-0680-3-8

**Published:** 2008-12-24

**Authors:** Georgii A Alexandrov, Tsuneo Matsunaga

**Affiliations:** 1 Office for Global Environmental Database, Center for Global Environmental Research, National Institute for Environmental Studies, Onogawa 16-2, Tsukuba, Japan; 2 Institute of Atmospheric Physics, Russian Academy of Sciences, Pyzhevsky 3, Moscow, Russia

## Abstract

**Background:**

The biosphere models of terrestrial productivity are essential for projecting climate change and assessing mitigation and adaptation options. Many of them have been developed in connection to the International Geosphere-Biosphere Program (IGBP) that backs the work of the Intergovernmental Panel on Climate Change (IPCC). In the end of 1990s, IGBP sponsored release of a data set summarizing the model outputs and setting certain norms for estimates of terrestrial productivity. Since a number of new models and new versions of old models were developed during the past decade, these normative data require updating.

**Results:**

Here, we provide the series of updates that reflects evolution of biosphere models and demonstrates evolutional stability of the global and regional estimates of terrestrial productivity. Most of them fit well the long-living Miami model. At the same time we call attention to the emerging alternative: the global potential for net primary production of biomass may be as high as 70 PgC y^-1^, the productivity of larch forest zone may be comparable to the productivity of taiga zone, and the productivity of rain-green forest zone may be comparable to the productivity of tropical rainforest zone.

**Conclusion:**

The departure from Miami model's worldview mentioned above cannot be simply ignored. It requires thorough examination using modern observational tools and techniques for model-data fusion. Stability of normative knowledge is not its ultimate goal – the norms for estimates of terrestrial productivity must be evidence-based.

## Background

The amount of plant organic matter produced on annual basis, so called net primary production or NPP, is the basic characteristic of the biosphere. It shows biosphere potential to supply primary food energy source for non-autotrophic species including humans. Human appropriation of terrestrial net primary production stems not only from the demand for food but also for fuel, construction materials, and paper. It is estimated to be from 8 to 15 PgC y^-1 ^in total (including 3–6 PgC y^-1 ^associated with food supply) [1].

NPP also shows biosphere potential to steer the Earth system by absorbing CO_2_, a gas whose atmospheric concentration affects global climate. NPP characterizes the "gross" terrestrial carbon sink – the amount of CO_2 _annually sequestered by vegetation. The net land-to-atmosphere flux is much smaller because the "gross" sink is compensated for by various carbon sources. Its magnitude is estimated to be from 0.3 to 1.5 PgC y^-1 ^[2]. The coupled carbon-cycle-climate models show the wide range of projections for the magnitude of the terrestrial uptake in the middle of this century: from 0 to 8 PgC y^-1 ^[3].

Appropriation (or re-direction) of NPP is also one method of climate change mitigation. Protecting non-living organic matter from decomposition and burning [4], reducing deforestation rates [5-7], and increasing the forest harvest age [8] will "re-direct" NPP to carbon pools with longer turnover times. Implementation of these measures may partly compensate for emissions from fossil fuel burning.

The total terrestrial NPP is generally assumed to be about 60 PgC y^-1 ^[9]. Biosphere models differ on this value. Comparison of global NPP models carried out more than a decade ago revealed that estimates ranged from 44.4 to 66.3 PgC y^-1 ^[10]. One of the major results of that comparative study was releasing average estimates of NPP over a geographic grid with a half-degree resolution [11]. These were the first normative data on global NPP created by summarizing modelling efforts. ("Normative data" means the data that result from a model ensemble, not from a single model, and therefore may be accepted as norms.)

The data have not been updated since then, although a number of new models and new versions of old models were developed during the last decade. Here we present the series of updates reflecting the evolution of biosphere models.

## Results

### Evolutional stability of normative data

The well-established beliefs in science tend to be evolutionarily stable – that is, new research on old subjects tends ultimately to furnish the same result. The series of updates (Additional files 1, 2, 3, 4, 5, 6, 7, 8, 9) demonstrates the evolutional stability of normative data on terrestrial productivity. The totals (i.e. the estimates of the total terrestrial NPP corresponding to different versions of the Normative NPP grid) vary in a very narrow range: from 58.76 to 59.14 PgC y^-1^. Sub-totals characterizing productivity of major vegetation zones are never off by more than 7 gC m^-2 ^y^-1 ^(Table 1). A new model may change sub-totals by 1% at most.

**Table 1:** Normative productivity of major vegetation zones

**Biome code**	**Normative NPP version**								
	
	**1.5**	**1.6**	**1.7**	**1.8**	**1.9**	**1.10**	**1.11**	**1.12**	**1.13**
**3**	792	795	796	793	788	787	787	787	792
**4**	812	816	818	815	813	815	814	815	817
**6**	64	63	62	62	62	63	62	62	60
**7**	318	319	319	319	318	320	318	318	321
**8**	995	1001	1002	999	1002	1008	1009	1008	1013
**10**	646	648	649	647	646	645	645	644	648
**13**	538	540	541	542	540	542	539	540	541
**14**	210	208	208	209	209	207	208	208	212
**15**	395	395	396	395	393	395	392	392	396
**27**	139	138	139	139	138	139	138	138	140
**36**	324	319	321	321	323	323	324	324	327
**42**	116	114	114	115	114	114	112	112	113

Units: gC m^-2 ^y^-1 ^(100 gC m^-2 ^y^-1 ^= tC ha^-1 ^y^-1^). Biome codes: 42 – tundra, 14 – larch forests, 36 – needle-leaf forests, 13 – summer-green broad-leaved forests, 4 – evergreen broad-leaved forests, 8 – tropical rainforests, 6 – deserts, 27 – semi-desert scrubs, 7 – shrublands, 15 – grasslands, 10 – subhumid woodlands, 3 – raingreen forests.

**Figure 1:**
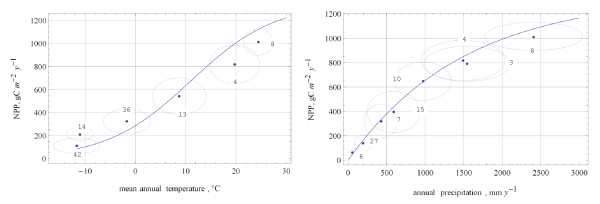
**Normative NPP (version 1.13.0) of major vegetation zones plotted against mean annual temperature (left pane) and annual precipitation (right pane)**. Points mark mean values, ellipses delineate standard deviations from the mean values, and lines represent temperature curve and humidity curve of the Miami NPP model, respectively. Legend: 42 – tundra, 14 – larch forests, 36 – needle-leaf forests, 13 – summer-green broad-leaved forests, 4 – evergreen broad-leaved forests, 8 – tropical rainforests, 6 – deserts, 27 – semi-desert scrubs, 7 – shrublands, 15 – grasslands, 10 – subhumid woodlands, 3 – raingreen forests.

**Figure 2:**
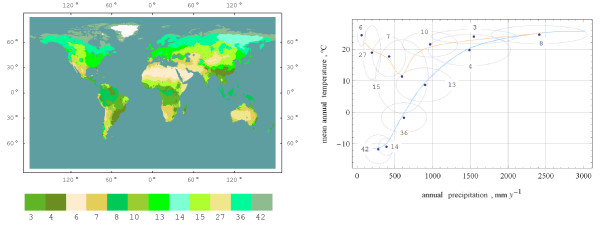
**The map (left pane) and climatic characteristics (right pane) of the vegetation zones**. Legend: 42 – tundra, 14 – larch forests, 36 – needle-leaf forests, 13 – summer-green broad-leaved forests, 4 – evergreen broad-leaved forests, 8 – tropical rainforests, 6 – deserts, 27 – semi-desert scrubs, 7 – shrublands, 15 – grasslands, 10 – subhumid woodlands, 3 – raingreen forests. Points mark mean values, ellipses delineate standard deviations from the mean values, and lines highlight ecological series (ecoclines). The blue line highlights the series of biomes succeeding each other along the gradient of mean annual temperature, and the red line the series of biomes succeeding each other along the gradient of annual precipitation. (The map of vegetation zones is based on the data from TGER data set [20], climatic characteristics are based on CLIMATE database version 2.1 [W. Cramer, Potsdam, personal communication].)

Most of sub-totals, in fact, fit well the "long-living" Miami NPP model (Figure 1). The Miami NPP model [12] is still used as a benchmark for NPP models and in global carbon cycle modelling [13-17]. Relating biome productivity to the mean annual temperature, this model implicitly presumes a certain correlation between the climatic conditions of the growing season and those of the whole year. Therefore, it may underestimate or overestimate productivity wherein the presumed correlation breaks down. For example, tundra (42) and the vegetation zone of larch forests (14) are equally cold in terms of mean annual temperature (Figure 2), but summer is warmer in the vegetation zone of larch forests. Therefore, process-based models, which are more sensitive to the seasonality of climatic conditions, normally estimate the productivity of larch forests to be higher than that of tundra. Similarly, they give higher estimate for the vegetation zone of needle-leaf evergreen forests (36). The lower estimate for tropical rainforests (8) may manifest the sensitivity of process-based models to limiting factors other than heat and water supply (e.g., nitrogen limitation).

### Emergent alternative data

**Figure 3:**
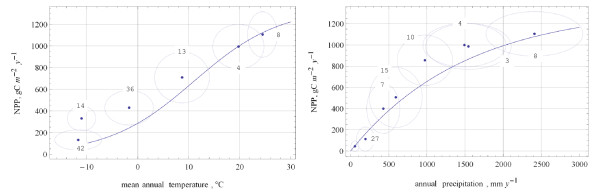
**Alternative NPP (version 1.13.0) of major vegetation zones plotted against mean annual temperature (left pane) and annual precipitation (right pane)**. Points mark mean values, ellipses delineate standard deviations from the mean values, and lines represent temperature curve and humidity curve of the Miami NPP model, respectively. Legend: 42 – tundra, 14 – larch forests, 36 – needle-leaf forests, 13 – summer-green broad-leaved forests, 4 – evergreen broad-leaved forests, 8 – tropical rainforests, 6 – deserts, 27 – semi-desert scrubs, 7 – shrublands, 15 – grasslands, 10 – subhumid woodlands, 3 – raingreen forests.

**Figure 4:**
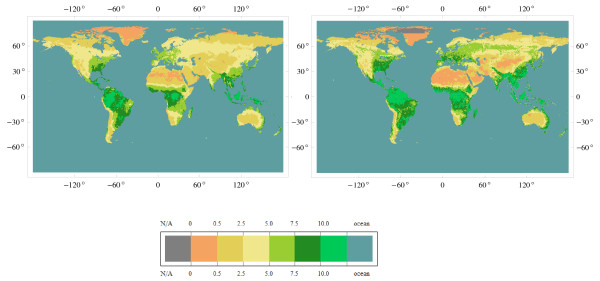
**Normative NPP version 1.13.0 (left pane) vs alternative NPP version 1.13.0 (right pane)**. Units: tC ha^-1 ^y^-1 ^(= 100 gC m^-2 ^y^-1^).

The method of building normative data employed in this study (see Methods) works against estimates suggesting too large shifts in mean values. These estimates form the pool of alternative data. The series of alternative data (Additional files 10, 11, 12, 13, 14, 15, 16, 17) shows large variations of totals: from 64 to 91.7 PgC y^-1^. In the final version (Additional file 17), the total is 71.4 PgC y^-1 ^and sub-totals depart widely from the Miami model projections (Figure 3) implying an alternative global pattern of productivity (Figure 4). This pattern may be characterized in general as "seasonality sensitive". The productivity of larch forests (14) is comparable to that of taiga (36), and the productivity of rain-green forests (3) is comparable to that of tropical rainforests (8), emphasizing that conditions during the growing season, not during the whole year, are crucial.

## Discussion

In modelling terrestrial productivity we are facing the problem of structural uncertainty. Field observations hardly allow us to make a reasonable choice between competing conceptual frameworks, to form an agreement on the best model structure, or even to discriminate between adequate descriptions of significant processes from inadequate ones [18]. Therefore, we approach this problem through retrospection of modelling efforts.

Terrestrial productivity has been a focus of biosphere studies over the last three decades. First, the global pattern of NPP was characterized by data collected during the International Biological Program (1964–1974). Then, the data was turned into empirical models that relate gradations in NPP to environmental factors of known geographic distribution. Later, a number of process-based models were developed in connection to the IGBP activities. This is definitely a field of science that hardly may be referred to as immature.

Nevertheless, the range of estimates remains roughly constant over this period. Early estimates of terrestrial NPP range from 10 to 100 PgC y^-1 ^[19]. Starting in the 1970s, they fall between 40 and 80 PgC y^-1^. The estimates of empirical models [20] vary from 50 to 65 PgC y^-1^, and the estimates of process-based models are expected to vary in the same range [10]. Re-analysis of the NPP measurements stored in the Osnabrück NPP database show that a 90% confidence interval for the expected value is 50–70 PgC y^-1 ^[21]. It seems that it may be difficult to reduce this 20% level of uncertainty in the commonly accepted estimate of terrestrial NPP while leaving research methods unchanged.

Therefore, we are focusing here on the stability of normative estimates – that is, estimates acceptable for use in policy relevant assessments. The diversity of research results does not matter until a viable alternative to the commonly accepted norms emerges. This study confirms that 60 PgC y^-1 ^remains to be the best candidate for further use in policy relevant assessments.

The major output of this study, gridded normative data on terrestrial productivity, may find use in benchmarking NPP models employed in coupled carbon-cycle-climate models. Could the wide range of projections for the future magnitude of the terrestrial uptake be attributed to the diversity of NPP models employed? Which projections correspond to well-established beliefs, and which do not?

Recent IPCC guidelines focus on objective reporting of uncertainty stemming from climate model pluralism [22]. However, epistemological pluralism [23,24] is no more a topical issue in "a world that is aware of its responsibility for planetary change and will demand globally concerted actions" [25,26]. One of the things that the world community is likely to expect from scientists is evaluating effectiveness of these actions in an objective and unambiguous manner [27]. Hence, it seems a time for moving the focus of attention to objective reporting of well-established beliefs.

Objective reporting of well-established beliefs suggests drawing distinctions between normative knowledge (or text-book knowledge) and alternative knowledge (or frontier knowledge). The former is the solid knowledge that has stood the test of time and is well confirmed by a number of independent research studies. Frontier knowledge is something new, and something really new cannot be turned into solid knowledge immediately. Since each model may be considered as normative for some regions and as alternative for other regions, we are not drawing distinctions between models. Instead, we are sorting model outputs and doing what is called "knowledge engineering" [28].

## Conclusion

The stability of well-established beliefs stems from the stability of research methods, and therefore it can be temporal. Rapid development of meteorological methods for measuring CO_2 _fluxes offers some benefits over traditional methods of measuring productivity. The observation network for measurements of gas, water and energy exchange between terrestrial ecosystems and the atmosphere, so-called FLUXNET [29], produced a large collection of data. This calls for re-calibration of existing models [30,31], and, hypothetically, may lead to changes in our judgement on typical values of productivity. Similar effects may have measurements coming from new satellite sensors.

**Figure 5:**
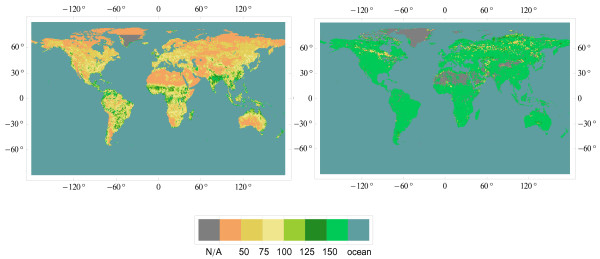
**The half-width of confidence interval for Normative NPP version 1.13.0 (left pane) vs that of alternative NPP version 1.13.0 (right pane)**. Units: gC m^-2 ^y^-1 ^(100 gC m^-2 ^y^-1 ^= tC ha^-1 ^y^-1^).

Modern observational tools will eventually improve the consistency of biosphere models through creating multiple constraints for positioning 'true' values for model parameters [32-35] and through filling gaps in knowledge needed for improving descriptions of significant processes. The evolution of normative knowledge on terrestrial productivity is thus limited by the rate at which the research community internalizes new facts and builds consensus on necessary changes in the norms. The scheme of building normative data [36] employed in this study simulates the process of consensus building, but sets transparent criteria for distinguishing between normative and alternative data. It also suggests that an existing consensus should be re-considered when the bulk and consistency of alternative data match the bulk and consistency of normative data (Figure 5). The software tools realizing this scheme are available through web-based services developed for routine checks of model consistency.

## Methods

The evolution of scientific theories is often considered a Darwinian process of natural selection that determines which theory survives and drifts them toward consensus [37]. The scheme of building normative data [36] employed in this study simulates the process of data selection by setting transparent criteria of fitness.

This algorithm works against new estimates that do not fall within the range implied by the initial ensemble: Miami NPP model, Montreal NPP model, TGER-NPP model and the outputs of the Potsdam NPP model intercomparison. Moreover, it works against new estimates that may increase uncertainty in the mean value, which is measured as the width of the confidence interval: *δ *= 2 *c*·*s*·*n*^-1/2^, where *n *is the number of estimates, *s *is standard deviation, *c *is 95th percentile of Student's *t *distribution with *n*-1 degree of freedom. All this is filtering out erroneous estimates as well as correct estimates when they are dramatically contradicting normative knowledge formed by the initial ensemble.

Since no well-agreed-upon method exists at the moment for distinguishing between erroneous and correct estimates of NPP, every estimate is included either into the normative ensemble or into the alternative ensemble. The former represents the current state of knowledge, whereas the latter represents emerging alternatives to current knowledge.

Noteworthy also are discrepancies in estimates that may result from different spatial resolution of models and/or input data. They were reduced by filtering out models of low spatial resolution.

### Normative NPP, version 1.5.0

This data file (Additional file 1) was formed by averaging the outputs of the Miami NPP model, Montreal NPP model, TGER-NPP model and the outputs of the Potsdam NPP model intercomparison (PotsdamNPP).

### Normative NPP, version 1.6.0

This data file (Additional file 2) was formed from the normative ensemble of estimates underlying Normative NPP 1.5.0. and outputs of TsuBiMo 1.0 model [18,38], using the following algorithm:

1. Compare the value of TsuBiMo NPP for a given cell (x,y) of the geographic grid, u(x,y) with the normative ensemble of estimates for this cell, w(x,y).

2. If u(x,y) > w_max_(x,y) or u(x,y) < w_min_(x,y), normative NPP, v(x,y), remains unchanged; otherwise go to step 3.

3. Append u(x,y) to w(x,y), calculate mean value, *μ*, of thus formed list of estimates and the width of its confidence interval, *δ*.

4. If *δ *is greater than the width of confidence interval for the mean value of w(x,y), v(x,y) remains unchanged, otherwise v(x,y) = *μ*.

NB. Exclude the numbers denoting missing values (-9999) from calculations of mean values and their confidence intervals.

### Normative NPP, versions 1.7.0–1.13.0

Each of the data files (Additional files 3, 4, 5, 6, 7, 8, 9) was formed, using the algorithm described above, from the normative ensemble of estimates and outputs of the model mentioned in the data file description.

### Alternative NPP, version 1.6.0

This data file (Additional file 10) was formed from the alternative ensemble of estimates underlying Alternative NPP version 1.5.0. and outputs of TsuBiMo 1.0 model, using the following algorithm.

1. Compare the value of TsuBiMo NPP for a given cell (x,y) of the geographic grid, u(x,y) with the normative ensemble of estimates (version 1.5.0) for this cell, w(x,y).

2. If w_min_(x,y) < u(x,y) < w_max_(x,y), alternative NPP, v(x,y), remains unchanged; otherwise go to step 3.

3. Append u(x,y) to w(x,y), calculate mean value of thus formed list of estimates and the width of its confidence interval, *δ*.

4. If *δ *is less than the width of confidence interval for the mean value of w(x,y), v(x,y) remains unchanged, otherwise go to the step 5.

5. Append u(x,y) to the list of alternative estimates, s(x,y); calculate the mean value, *μ*, of thus formed list; set v(x,y) = *μ*.

NB. Exclude the numbers denoting missing values (-9999) from calculations of mean values and their confidence intervals.

### Alternative NPP, versions 1.7.0–1.13.0

Each of the data file (Additional files 11, 12, 13, 14, 15, 16, 17) was formed, using the algorithm described above, from the alternative ensemble of estimates and outputs of the model mentioned in the data file description.

## Warnings

The results of this study should be interpreted in the same manner as the results of Potsdam NPP Model Intercomparison [10], and should not be taken out of context. For example, normative productivity of crops under specific crop management system may be either higher or lower than Normative NPP.

## Competing interests

The authors declare that they have no competing interests.

## Authors' contributions

The authors contributed equally to the design of the study and to manuscript drafting. Calculations were performed by GAA. Both authors read and approved the final version of the manuscript.

## Appendix: a brief overview of modelling efforts

Efforts to model terrestrial productivity may be categorized into three types: (type 1) developing an empirical model interpolating and extrapolating measured NPP values, (type 2) developing an empirical model interpolating and extrapolating parameters of a process-based model of productivity using measured NPP values, (type 3) developing an empirical model interpolating and extrapolating parameters of a process-based model of productivity using measured values of this parameters.

The typical representative of the first type, which is referred to as empirical models, is the Miami NPP model. The observed gradations in the observed data are attributed to factors of known global distribution using mathematical functions that have no biological meaning. Then, these functions are used to produce a global pattern of productivity from given global patterns of mean annual temperature and precipitations.

TsuBiMo represents the second type, which is referred to as semi-empirical process-based models. The measured values of NPP are considered as indirect measurements of light-saturated rates of photosynthesis [18]. The values of this parameter are restored from the NPP measurements using technique known as model-data fusion [39]. Then, gradations in the light-saturated rate of photosynthesis are attributed to gradations in the temperature and precipitation during the growing season. The temperature dependence is modelled with a generalized Arrhenius function [40], whereas the humidity factor is modelled with a function that has no biophysical meaning.

Biome-BGC represents the third type, which is referred to as process-based models, and GLO-PEM represent a class of so-called production efficiency models (PEMs). A number of such models were developed between 1992 and 1996 (Table 2). The boom has been backed by supportive databasing activities. In 1991, the International Institute for Applied System Analysis (IIASA) released the database for mean monthly values of temperature, precipitation and cloudiness of a global terrestrial grid [41]. In 1992, the Environmental Research Laboratory of the US Environmental Protection Agency released a comprehensive geographic database for modelling terrestrial climate-biosphere interactions [42]. Data on the global distribution of productivity factors stimulated globalization of process-based models that were originally developed for modelling productivity at an ecosystem scale [43,44].

**Table 2:** A chronology of modelling efforts

**Year**	**Type 1**	**Type 2**	**Type 3**
**1972**	Miami NPP** [12]; Montreal NPP** [54]		

		**1973 ... 1984**	

**1985**	Chikugo [55,56]		

**1986**			

**1987**	OBM [13]		

**1988**	MONTHLYC [57]		

**1989**	TGER-NPP** [20,58]		

**1990**			

**1991**			

**1992**			

**1993**			Biome-BGC [44]; CASA [59]; FBM 2.2 [60]; CENTURY 4.0 [61]; TEM 3.0 [62]

**1994**	HRBM 3.0 [63]		CARAIB 2.1 [64]; DEMETER [65]; IMAGE 2.0 [66]

**1995**			GLO-PEM [67]; SDBM[68]; TEM 4.0 [69];

**1996**			DOLY [70]; SIB2 [71]; SILVAN 2.2 [72]; TURC [73]; BIOME3 [74]

**1997**			HYBRID 3.0 [75]; BAIM [76,77]

**1998**		TsuBiMo 1.0** [38,78]	

**1999**			GLO-PEM updated version *,** [79]

**2000**			Sim-Cycle** [80]; BETHY [81,82]

**2001**			

**2002**		TsuBiMo-PEM [18]; TsuBiMo 1.1 [18];	CCDAS-SDBM [83]

**2003**			

**2004**		TsuBiMo 1.2 [84]	MODIS-NPP*,** [85,86]; Sim-Cycle(rev)** [87]

**2005**		TsuBiMo 1.3 [88]	BEAMS** [89]; VEGAS** [90,91]

**2006**			BAIM2 [92]; ORCHIDEE[93]; CCDAS-BETHY [94,95]

**2007**			Biome-BGC 4.1.1** [96]; JSBACH [97]

**2008**	NCEAS [45]; Madison NPP** [98]		

The list includes only those models of productivity that were calibrated for global scale applications of fine spatial resolution (1-degree or higher). The series of models developed in 80s [46-50] was not included, for their spatial resolution is too coarse (the biosphere is split into several discrete productivity zones.) Some dynamic global vegetation models [33,51] were not included in the list either because we did not receive confirmation from the authors that model outputs include an original baseline annual estimate of productivity or because of the coarse resolution of the model product [52]. Any corrections and suggestions to this list can be posted as a comment to the article, pressing "Post a Comment" button on the article website.

* model outputs are available for downloading from a public website

** model outputs are stored in Carbon Sink Archives and available for model benchmarking using the web-based service provided by the OGED/CGER/NIES [53]

Most process-based models require species-specific parameterization that becomes problematic on a global scale. Since the global distribution of species-specific parameters is not well known, they are normally set at some ad hoc values. The recent development of techniques for model-data fusion [34] opens up possibilities for transforming process-based models into semi-empirical, process-based models.

New techniques for data-fusion (such as neural networks) together with growing databases of NPP measurements offer new opportunities for empirical modelling. The NCEAS model [45] may be a first sign of the new boom in empirical modelling.

The history of modelling efforts is presented in Table 2, where models [44-98] are listed in chronological order.

## Supplementary Material

Additional file 1 **Gridded normative NPP, version 1.5.0.** The file can be viewed with a spreadsheet. It stores tabular data: 3 columns separated by tabs and 62483 rows. Each row corresponds to a cell of the geographic grid of half-degree resolution. First two columns contain longitude and latitude for the north-west corner a cell in decimal degrees. West longitudes and south latitudes are negative. The third column contains NPP estimate in gC m^-2 ^y^-1 ^obtained by averaging outputs of Miami NPP model, Montreal NPP model, TGER-NPP model and the outputs of the Potsdam NPP model intercomparison (PotsdamNPP). Missing values are denoted by -9999. Click here for file

Additional file 2 **Gridded normative NPP, version 1.6.0.** The file can be viewed with a spreadsheet. It stores tabular data: 3 columns separated by tabs and 62483 rows. Each row corresponds to a cell of the geographic grid of half-degree resolution. First two columns contain longitude and latitude for the north-west corner a cell in decimal degrees. West longitudes and south latitudes are negative. The third column contains NPP estimate in gC m^-2 ^y^-1 ^formed from the normative ensemble of estimates underlying Normative NPP 1.5.0. and outputs of TsuBiMo 1.0 model. Missing values are denoted by -9999. Click here for file

Additional file 3 **Gridded normative NPP, version 1.7.0.** The file can be viewed with a spreadsheet. It stores tabular data: 3 columns separated by tabs and 62483 rows. Each row corresponds to a cell of the geographic grid of half-degree resolution. First two columns contain longitude and latitude for the north-west corner a cell in decimal degrees. West longitudes and south latitudes are negative. The third column contains NPP estimate in gC m^-2 ^y^-1 ^formed from the normative ensemble of estimates underlying Normative NPP 1.6.0. and outputs of the updated version of GLO-PEM. Missing values are denoted by -9999. Click here for file

Additional file 4 **Gridded normative NPP, version 1.8.0.** The file can be viewed with a spreadsheet. It stores tabular data: 3 columns separated by tabs and 62483 rows. Each row corresponds to a cell of the geographic grid of half-degree resolution. First two columns contain longitude and latitude for the north-west corner a cell in decimal degrees. West longitudes and south latitudes are negative. The third column contains NPP estimate in gC m^-2 ^y^-1 ^formed from the normative ensemble of estimates underlying Normative NPP 1.7.0. and outputs of Biome-BGC 4.1.1. Missing values are denoted by -9999. Click here for file

Additional file 5 **Gridded normative NPP, version 1.9.0.** The file can be viewed with a spreadsheet. It stores tabular data: 3 columns separated by tabs and 62483 rows. Each row corresponds to a cell of the geographic grid of half-degree resolution. First two columns contain longitude and latitude for the north-west corner a cell in decimal degrees. West longitudes and south latitudes are negative. The third column contains NPP estimate in gC m^-2 ^y^-1 ^formed from the normative ensemble of estimates underlying Normative NPP 1.8.0. and outputs of BEAMS. Missing values are denoted by -9999. Click here for file

Additional file 6 **Gridded normative NPP, version 1.10.0.** The file can be viewed with a spreadsheet. It stores tabular data: 3 columns separated by tabs and 62483 rows. Each row corresponds to a cell of the geographic grid of half-degree resolution. First two columns contain longitude and latitude for the north-west corner a cell in decimal degrees. West longitudes and south latitudes are negative. The third column contains NPP estimate in gC m^-2 ^y^-1 ^formed from the normative ensemble of estimates underlying Normative NPP 1.9.0. and outputs of Madison NPP model. Missing values are denoted by -9999. Click here for file

Additional file 7 **Gridded normative NPP, version 1.11.0.** The file can be viewed with a spreadsheet. It stores tabular data: 3 columns separated by tabs and 62483 rows. Each row corresponds to a cell of the geographic grid of half-degree resolution. First two columns contain longitude and latitude for the north-west corner a cell in decimal degrees. West longitudes and south latitudes are negative. The third column contains NPP estimate in gC m^-2 ^y^-1 ^formed from the normative ensemble of estimates underlying Normative NPP 1.10.0. and outputs of Improved MODIS Collection 4.8 NPP. Missing values are denoted by -9999. Click here for file

Additional file 8 **Gridded normative NPP, version 1.12.0.** The file can be viewed with a spreadsheet. It stores tabular data: 3 columns separated by tabs and 62483 rows. Each row corresponds to a cell of the geographic grid of half-degree resolution. First two columns contain longitude and latitude for the north-west corner a cell in decimal degrees. West longitudes and south latitudes are negative. The third column contains NPP estimate in gC m^-2 ^y^-1 ^formed from the normative ensemble of estimates underlying Normative NPP 1.11.0. and outputs of Sim-CYCLE(rev). Missing values are denoted by -9999. Click here for file

Additional file 9 **Gridded normative NPP, version 1.13.0.** The file can be viewed with a spreadsheet. It stores tabular data: 3 columns separated by tabs and 62483 rows. Each row corresponds to a cell of the geographic grid of half-degree resolution. First two columns contain longitude and latitude for the north-west corner a cell in decimal degrees. West longitudes and south latitudes are negative. The third column contains NPP estimate in gC m^-2 ^y^-1 ^formed from the normative ensemble of estimates underlying Normative NPP 1.12.0. and outputs of VEGAS. Missing values are denoted by -9999. Click here for file

Additional file 10 **Gridded alternative NPP, version 1.6.0.** The file can be viewed with a spreadsheet. It stores tabular data: 3 columns separated by tabs and 62483 rows. Each row corresponds to a cell of the geographic grid of half-degree resolution. First two columns contain longitude and latitude for the north-west corner a cell in decimal degrees. West longitudes and south latitudes are negative. The third column contains NPP estimate in gC m^-2 ^y^-1 ^formed from the alternative ensemble of estimates underlying Alternative NPP version 1.5.0. and outputs of TsuBiMo 1.0. Missing values (i.e., the lack of alternative estimate) are denoted by -9999. Click here for file

Additional file 11 **Gridded alternative NPP, version 1.7.0**. The file can be viewed with a spreadsheet. It stores tabular data: 3 columns separated by tabs and 62483 rows. Each row corresponds to a cell of the geographic grid of half-degree resolution. First two columns contain longitude and latitude for the north-west corner a cell in decimal degrees. West longitudes and south latitudes are negative. The third column contains NPP estimate in gC m^-2 ^y^-1 ^formed from the alternative ensemble of estimates underlying Alternative NPP version 1.6.0. and outputs of the updated version of GLO-PEM. Missing values (i.e., the lack of alternative estimate) are denoted by -9999. Click here for file

Additional file 12 **Gridded alternative NPP, version 1.8.0.** The file can be viewed with a spreadsheet. It stores tabular data: 3 columns separated by tabs and 62483 rows. Each row corresponds to a cell of the geographic grid of half-degree resolution. First two columns contain longitude and latitude for the north-west corner a cell in decimal degrees. West longitudes and south latitudes are negative. The third column contains NPP estimate in gC m^-2 ^y^-1 ^formed from the alternative ensemble of estimates underlying Alternative NPP version 1.7.0. and outputs of Biome-BGC 4.1.1. Missing values (i.e., the lack of alternative estimate) are denoted by -9999. Click here for file

Additional file 13 **Gridded alternative NPP, version 1.9.0.** The file can be viewed with a spreadsheet. It stores tabular data: 3 columns separated by tabs and 62483 rows. Each row corresponds to a cell of the geographic grid of half-degree resolution. First two columns contain longitude and latitude for the north-west corner a cell in decimal degrees. West longitudes and south latitudes are negative. The third column contains NPP estimate in gC m^-2 ^y^-1 ^formed from the alternative ensemble of estimates underlying Alternative NPP 1.8.0. and outputs of BEAMS. Missing values are denoted by -9999. Click here for file

Additional file 14 **Gridded alternative NPP, version 1.10.0.** The file can be viewed with a spreadsheet. It stores tabular data: 3 columns separated by tabs and 62483 rows. Each row corresponds to a cell of the geographic grid of half-degree resolution. First two columns contain longitude and latitude for the north-west corner a cell in decimal degrees. West longitudes and south latitudes are negative. The third column contains NPP estimate in gC m^-2 ^y^-1 ^formed from the alternative ensemble of estimates underlying Alternative NPP 1.9.0. and outputs of Madison NPP model. Missing values are denoted by -9999. Click here for file

Additional file 15 **Gridded alternative NPP, version 1.11.0.** The file can be viewed with a spreadsheet. It stores tabular data: 3 columns separated by tabs and 62483 rows. Each row corresponds to a cell of the geographic grid of half-degree resolution. First two columns contain longitude and latitude for the north-west corner a cell in decimal degrees. West longitudes and south latitudes are negative. The third column contains NPP estimate in gC m^-2 ^y^-1 ^formed from the alternative ensemble of estimates underlying Alternative NPP 1.10.0. and outputs of Improved MODIS Collection 4.8 NPP. Missing values are denoted by -9999. Click here for file

Additional file 16 **Gridded alternative NPP, version 1.12.0.** The file can be viewed with a spreadsheet. It stores tabular data: 3 columns separated by tabs and 62483 rows. Each row corresponds to a cell of the geographic grid of half-degree resolution. First two columns contain longitude and latitude for the north-west corner a cell in decimal degrees. West longitudes and south latitudes are negative. The third column contains NPP estimate in gC m^-2 ^y^-1 ^formed from the alternative ensemble of estimates underlying Alternative NPP 1.11.0. and outputs of Sim-CYCLE2. Missing values are denoted by -9999. Click here for file

Additional file 17 **Gridded alternative NPP, version 1.13.0.** The file can be viewed with a spreadsheet. It stores tabular data: 3 columns separated by tabs and 62483 rows. Each row corresponds to a cell of the geographic grid of half-degree resolution. First two columns contain longitude and latitude for the north-west corner a cell in decimal degrees. West longitudes and south latitudes are negative. The third column contains NPP estimate in gC m^-2 ^y^-1 ^formed from the alternative ensemble of estimates underlying Alternative NPP 1.12.0. and outputs of VEGAS. Missing values are denoted by -9999. Click here for file
